# Stem Cell-Like Circulating Tumor Cells Indicate Poor Prognosis in Gastric Cancer

**DOI:** 10.1155/2014/981261

**Published:** 2014-05-22

**Authors:** Man Li, Baogang Zhang, Zhiguang Zhang, Xia Liu, Xiangjuan Qi, Jianqiu Zhao, Yong Jiang, Haoyu Zhai, Yinglan Ji, Dan Luo

**Affiliations:** ^1^Department of Digestive, The Second Hospital of Tianjin Medical University, Tianjin 300211, China; ^2^Tianjin Children Hospital, Tianjin 300074, China

## Abstract

Circulating tumor cells (CTCs), which have stem cell-like characteristics, might play a crucial role in cancer metastasis. CD44 has been identified as gastric cancer (GC) stem cell (CSC) marker. Here, the prognostic significance of CD44-positive CTCs in GC patients was investigated. CTCs were detected in 27 of 45 GC patients. The presence of CTCs was significantly associated with lymph node metastasis, distant metastasis, and recurrence (*P* = 0.007, *P* = 0.035, and *P* = 0.035, resp.). Nineteen of the 27 CTC-positive patients had CD44-positive CTCs. These patients were more likely to develop metastasis and recurrence than patients with CD44-negative CTCs. CD44-positive CTC counts were higher in recurrent patients than in the nonrecurrent ones (means 4.8 and 1.9, resp.; *P* = 0.010). Furthermore, 13 of 19 patients with CD44-positive CTCs developed recurrent disease, and the mean time to recurrence was shorter than that in patients with CD44-negative CTCs (10.54 ± 5.55 and 19.13 ± 9.72 months, resp.; *P* = 0.04). COX proportional hazards model indicated that the presence of CD44-positive CTCs and TNM stage were independent predictors of recurrence for GC (*P* = 0.030 and 0.008). So identifying the stem cell-like CTC subset may provide more clinically useful prognostic information than only detecting CTCs.

## 1. Introduction

Gastric cancer (GC) is the second leading cause of cancer-related deaths in the world [1]. Despite advances in diagnostic tools and therapeutic methods, the 5-year relative survival rate is still less than 30% [2]. Common causes of death in GC patients are recurrent and metastatic disease.

Recently, many studies in medical science have focused on disseminated tumor cells present in patients' blood, known as circulating tumor cells (CTCs), which correlate with the risk of recurrence and metastasis in cancer patients. CTCs could be detected in cancer patients with no clinically detectable metastasis and the presence of CTCs is associated with poor patient prognosis [3, 4]. However, not all CTCs have the potency to develop into metastasis. Only the small population of CTCs with stem cell-like properties can survive and migrate to distant sites to establish secondary tumors. These cells are called circulating tumor stem cells (CTSC) and have the ability to self-renew, proliferate, and initiate tumors similar to cancer stem cells (CSCs) [5].

Recent advances in technology have allowed the detection and characterization of CTCs in GC. It has been shown that presence of CTCs is an independent predictive marker of poor prognosis in GC patients [6]. However, no studies investigating the prognostic and biological relevance of CTSC in GC patients have been reported. In previously published work, CD44-positive GC cells were highly invasive and exhibited the stem cell property of self-renewal [7]. Thus, CD44 might be served as a biomarker for tumor-initiating cells in GC.

Here, we identified GC patients with CD44-positive CTCs and evaluated their clinical characteristics to test the hypothesis that these represent more aggressive stem cell-like subpopulation of CTCs in GC.

## 2. Materials and Methods

### 2.1. Tissue Specimens

Blood samples from 45 GC patients being treated at the 2nd Hospital of Tianjin Medical University from March 2010 to December 2012 were obtained before the initiation of treatment. Detailed clinicopathological data, including age, gender, tumor location, TNM stage, and distant and lymph node metastasis, were collected by reviewing medical charts and pathological records for all of the patients. Clinical outcome was followed from the date of diagnosis until December 2013. The diagnoses of recurrence and metastasis were based on the computed tomography scans, with or without histological confirmation. Blood samples from 20 healthy volunteers acted as controls. The study was approved by the local ethics committee. Informed consent was obtained from both GC patients and cancer-free volunteers before obtaining samples.

### 2.2. Sample Preparation

Approximately 10 mL blood was collected in EDTA vacutainer tubes after discarding the first 2 mL of blood to avoid contamination of the blood sample with epithelial cells of skin. Peripheral blood mononuclear cells (PBMCs) were isolated by density gradient centrifugation using Lymphocyte Separation Medium (Tianjin Chuanye Biochemical Co., Ltd.). The mononuclear cells then were washed twice with 1 × phosphate buffered saline (PBS) and centrifuged at 1800 rpm for 10 min. The cells were resuspended in 100 *μ*L 1 × PBS and spread onto glass slides using a cytocentrifuge, prior to being fixed with 4% methanol. Fixed slides were stored at −80°C until use. Two slides from each patient were used for staining experiments.

The human gastric cancer cell line BGC-823 was obtained from the Tianjin Cancer Research Institute. To determine assay sensitivity, 10 BGC-823 cells were added to 10 mL of blood from healthy volunteersprior to being processed as described above for patients' samples.

### 2.3. Double Immunofluorescent Staining to Detect CK19 and CD44

Cytospin preparations were washed in 1 × PBS at room temperature for 15 min and permeabilized with Triton X-100. Following blocking with undiluted normal goat serum for 1 h, the samples were incubated with CK19 rabbit polyclonal antibody (dilution 1 : 100, BA2266-1, Boster, China) and Leukocyte Common Antigen (LCA/CD45, mouse monoclonal antibody, dilution 1 : 100, ZM-0183, Zhongshan, China) for 1 h and washed for 10 min with blocking buffer, followed by incubation with CD44 mouse monoclonal antibody (sc-65265, Santa Cruz) diluted 1 : 100 in a dark, humid chamber to stay overnight. After at least 20 hours, cytospin preparations were washed in 1 × PBS at room temperature for 15 min again. The samples were incubated with FITC-conjugated rabbit anti-human IgG (dilution 1 : 100, ZF0306, Zhongshan, China) and Rhodamine (TRITC)-conjugated AffiniPure Goat Anti-mouse IgG (dilution 1 : 100, ZF0313, Zhongshan, China) for 60 min. Cells were postfixed with methanol for 5 min at −20°C, washed twice with 1 × PBS, and DNA stained with 4,6-diamino-2-phenylindole (DAPI). The samples were washed twice with PBS and mounted with coverslips.

The cytomorphological criteria proposed by Meng and colleagues [8] (e.g., high nuclear/cytoplasmic ratio and cells larger than white blood cells) were used to characterize a CK19-positive cell as a CTC. CTC-positive case was defined by the presence of at least 1 CTC per 10 mL sample.

CK19 and/or CD44 expression was analyzed by a computerized fluorescence microscope (Imager 090–135.001, Leica, Germany). Samples were visualized by light microscopy to identify CK19-positive-cells (green staining). After detection of CK19-positive cells, samples were analyzed by fluorescence microscopy to identify CD44-positive cells (red staining).

Five fields with the greatest number of CK19-positive CTCs or CD44/CK19 double positive CTCs were chosen to quantify the number of CTCs in the sample. The average count of five fields at 400x magnification was recorded as the mean count of CTCs (CK19-positive only) or CD44-positive CTCs (double CK19/CD44-positive cells).

### 2.4. Statistical Methods

Statistical analysis was conducted using the SPSS 16.0 software (SPSS, Chicago, IL, USA). Data are expressed either as mean ± standard deviation or as percentages. The *χ* ^2^ test, the Student's *t*-test, and Mann Whitney test were used to establish significance. Two-tailed *P* < 0.05 values were considered statistically significant. Kaplan-Meier survival analysis and log-rank test were performed to analyze the time to recurrence for the CTCs and CD44-positive CTCs groups. Multivariate recurrence analysis was performed using the COX proportional hazards model.

## 3. Results

### 3.1. Patient Characteristics

45 GC patients, comprising 27 males and 18 females, with a mean age of 62.18 ± 10.57 years were included in the present study. The time to recurrence ranged from 1 to 45 months (mean 15.12 ± 9.03 months). Of the 45 patients, 25 had TNM stage I/II and 20 had TNM stage III/IV.

### 3.2. Definition of CTCs and CD44-Positive CTCs in GC Patients

**Figure 1:**
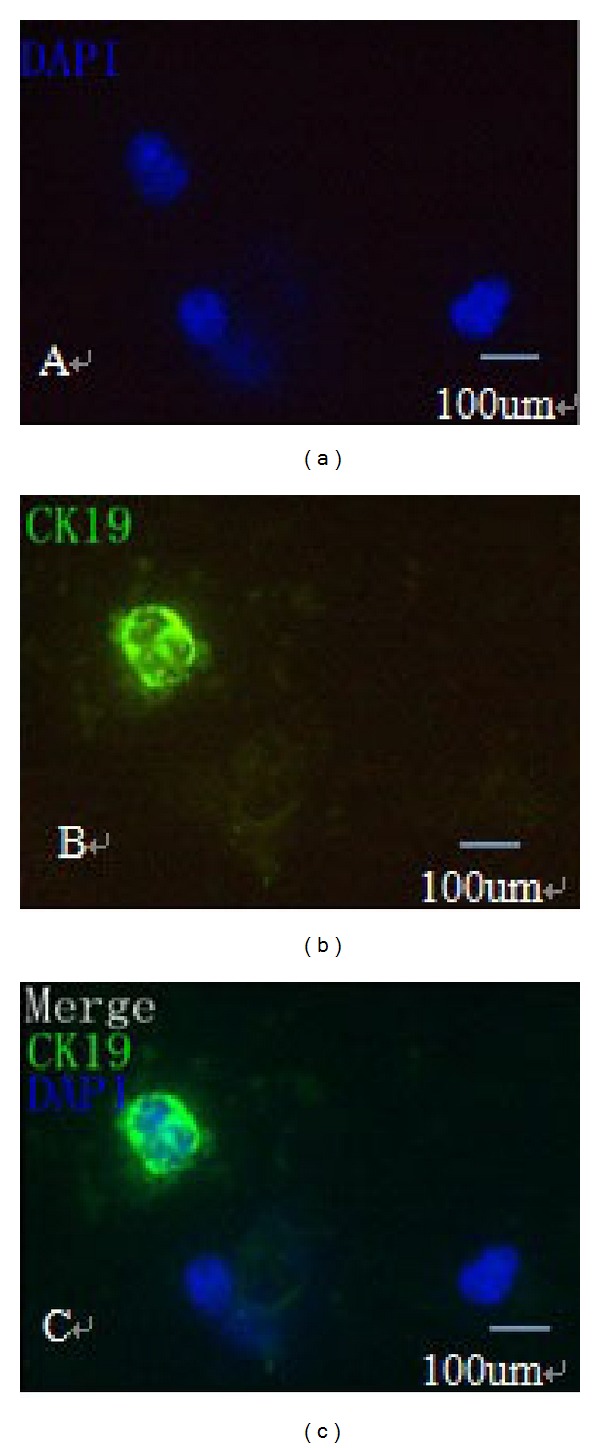
Detection of CTCs using immunofluorescent staining (×400). (a) Chromatin was decorated by the DNA-binding dye DAPI (blue staining). (b) CK19 was green staining. (c) A CTC was identified by CK19-positive staining with high nuclear/cytoplasmic ratio and cells larger than white blood cells.

**Figure 2:**
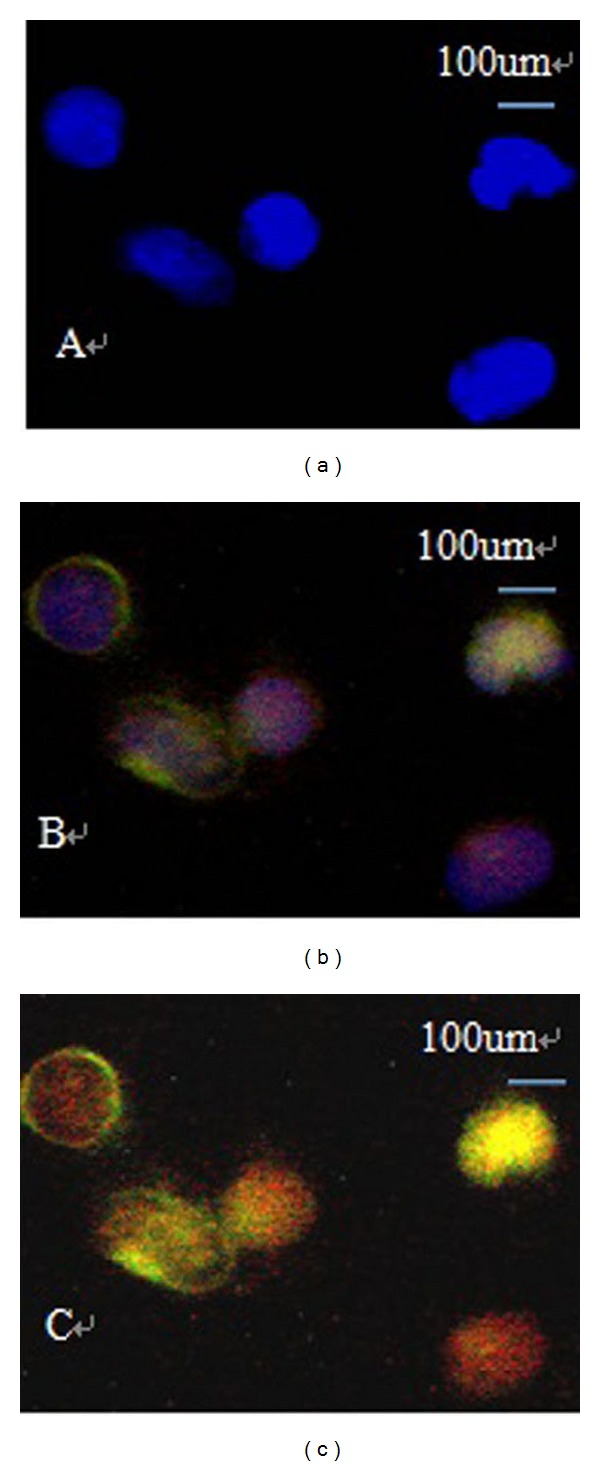
Detection of CD44-positive CTCs using double-immunofluorescent staining (×400). (a) Chromatin was decorated by the DNA-binding dye DAPI (blue staining). (b) CK19 was green staining. (c) CD44 (red) and CK19 (green) were costained.

A methodology that would permit specific double staining for CK19 and CD44, as well as DNA, in the same sample was established. Prior to this, we added 10 BGC-823 human gastric cancer cells into 10 mL blood obtained from healthy volunteers. In these spike-recovery experiments, the recovery rate of BCG-823 cells was approximately between 65% and 75%. CTCs were defined as nucleated intact cells that were positive for CK19 and negative for Leukocyte Common Antigen (LCA/CD45) (Figure 1). CK19-positive cells, defined here as CTCs, were further evaluated for the expression of CD44. Cells with CK19/CD44 both staining were defined as CD44-positive CTCs (Figure 2).

### 3.3. Relationships between CTCs and Clinicopathological Features of GC Patients

Detailed clinicopathological data and CTC status in the GC patients included in this study are summarized in Table 1. The presence of CTCs was significantly associated with lymph node metastasis, distant metastasis, and recurrence of GC (*P* = 0.007, *P* = 0.035, and *P* = 0.035, resp.). Of the 45 GC patients analyzed, 27 (60.0%) were identified as CTC positive. Among the 27 CTC-positive patients, 19 patients (70.4%) and 15 patients (55.6%) developed lymph node and distant metastasis, respectively. Recurrence was observed in 15 of the 27 (55.6%) CTC-positive patients. No statistically significant difference was detected between the CTC-positive patients and the CTC-negative patients with respect to age, gender, tumor location, and TNM stage.

### 3.4. Relationships between CD44-Positive CTCs and Clinicopathological Features of GC Patients

**Figure 3:**
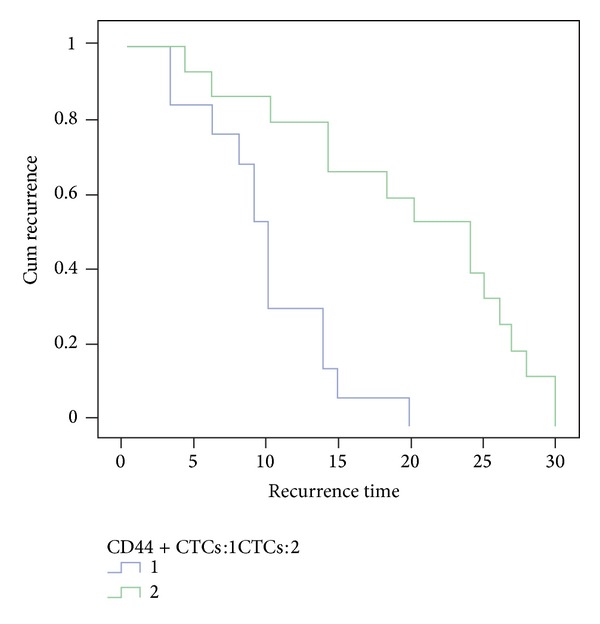
The recurrent time of CD44-positive CTCs group was shorter than that of CTCs group.

Clinicopathological data and CD44-positive CTC status in the GC patients included in this study are summarized in Table 2. The incidence of CD44-positive CTCs was significantly associated with tumor location, lymph node metastasis, distant metastasis, and recurrence of GC (*P* = 0.027, *P* = 0.033, *P* = 0.033 and *P* = 0.011, resp.). CD44-positive CTCs were detected in 19 (70.4%) of the 27 CTC-positive patients (Table 1). Among the 19 patients with CD44-positive CTCs, 14 patients (73.7%) and 12 patients (63.2%) developed lymph node and distant metastasis, respectively. Of 27 patients with recurrent disease, 14 were positive for CTCs and 13 of the CTC-positive patients were also positive for CD44-positive CTCs. The number of CD44-positive CTCs was significantly higher in patients with recurrent disease than in disease free patients (mean number of CD44-positive CTC 4.8 and 1.9, resp.; *P* = 0.010). Furthermore, of the 19 patients that had CD44-positive CTCs, 13 patients (68.4%) developed recurrent disease (mean time to recurrence 10.54 ± 5.55 months) which was significantly shorter than the time to recurrence in the CTC-positive patients (mean time to recurrence 19.13 ± 9.72 months; *P* = 0.04; Figure 3). Finally, patients with gastric cardia cancer were more likely to have CD44-positive CTCs than gastric noncardia cancer patients (16 out of 19 patients and 3 out of 8 patients, resp.; *P* = 0.027). Cox proportional hazards model analysis was performed and showed that the presence of CD44-positive CTCs and the TNM stage were independent indicators of recurrence for GC (*P* = 0.030 and 0.008).

## 4. Discussion

Recently, circulating tumor cells (CTCs) have emerged as an important field of study in biomedical research. Detection of CTCs is an early marker of tumor recurrence occurring before clinical symptoms present. CTCs quantitation could serve as a “liquid biopsy” to predict poor prognosis in a number of epithelial-derived cancers [9–11].

Most recent studies investigating CTCs have been carried out in breast cancer. There have been fewer studies focusing on CTCs in gastric cancer and the results from these studies have been inconsistent [12].

Biotechnology advances have allowed the detection and characterization of CTCs in cancer patients. Because CTCs occurat very low numbers, a blood volume of at least 7.5 mL is generally required for analysis. In general, analysis of CTCs involves primary enrichment followed by CTC detection. Enrichment may be achieved using various methodologies based on physical properties and biological properties of CTCs which differentiate them from the normal blood cells [13]. In our research, density gradient centrifugation was used to enrich CTCs. Immunofluorescence was used to identify CTCs, which were defined as CK19 positive (a marker for epithelial tumor cells) and CD45 negative (the common leukocyte antigen), while DNA was detected with DAPI. These approaches have been proven to be both sensitive and effective [14].

Gastric cancer is the most frequent malignancy in the world. Although many advances have been made in the early diagnosis and surgical treatment, patient prognosis remains poor. The major cause of death from GC is the inability to detect and prevent metastasis at an early stage of disease. Several studies investigating the presence of CTCs in gastric cancer patients have been reported in the literature, but both the CTC detection techniques used and the results obtained were heterogeneous [15–17]. And most of these studies focused on the sensitivity and specificity of CTCs isolation, as well as the relationship between CTCs and cancer relapse.

CTCs could be regarded as progenitors of cancer relapse. However, the presence of CTCs in the circulation is not sufficient to initiate metastasis because only a minority of the CTCs may possess the stem cell-like properties required to reseed or metastasize to distant organs. This has led to the hypothesis that CTCs may have the hallmarks of cancer stem cells (CSCs) which could allow them to form a tumor at a distant site. Recent studies have demonstrated that stem cell markers are frequently overexpressed in CTCs [18, 19]. Sun et al. have reported that the presence of circulating stem cell-like epithelial cell adhesion molecule-positive (EpCAM) tumor cells is associated with a poor prognosis for hepatocellular carcinoma patients following curative resection [20].

Therefore, we hypothesized that identifying the stem cell-like CTC subpopulation would provide more prognostic information than CTC quantitation alone. In our present study, we tested whether CTCs with CSCs properties play a crucial role in the spread of cancer in GC patients.

Zhang et al. and Takaishi et al. have demonstrated that CD44-positive gastric cancer cells exhibit the properties of self-renewal and the ability to produce differentiated progeny, both of which are consistent with a CSC phenotype [7, 21]. Accordingly, we used CD44 as a putative stem cell marker to identify CTSC in this study.

In our prospective study of 45 GC patients, we found that the presence of CTCs was correlated with tumor metastasis and recurrence. 27 of 45 GC patients were identified as CTCs positively by immunofluorescent technology. Among them, 19 and 15 patients have developed lymph node and distant metastasis, respectively. Recurrence was observed in 15 of 27 CTCs-positive patients. This data suggest that the presence of CTCs is a potential indicator of poor prognosis for GC patients. These results are in agreement with other reports in the literature for both gastrointestinal and other [22, 23]. Based on these results, we carried out double immunofluorescent staining to determine if CD44-positive CTCs, with a more stem cell-like phenotype, represent a more aggressive subset of CTCs. The presence of CTCs is known to be necessary but not sufficient for the initiation of metastasis because only a minority cells possess the stem cell-like properties necessary to survive and reseed a tumor at a distant site. Thus, detection of CD44-positive CTCs might serve as a novel marker for clinically undetectable metastases and recurrence risk for GC patients. Our data indicate that patients with CD44-positive CTCs were more likely to develop metastases and experience disease recurrence than patients with only CTCs. Our results show that CD44-positive CTCs were identified in 19 of 27 CTCs-positive patients. Those subpopulations were easily to develop metastasis and recurrence than that only CTCs-positive group. The incidence of lymph node metastasis, distant metastasis, and recurrence for two groups were shown as follows: 14/19 (73.68%) versus 19/27 (70.37%), 12/19 (63.16%) versus 15/27 (55.56%), and 13/19 (68.42) versus 15/27 (55.56%), respectively. In addition, the mean time to recurrence was shorter in patients with CD44-positive CTCs. Additionally, we found that CD44-positive CTCs were more often detected in patients with gastric cardia cancer. This could explain the more severe malignancy observed in gastric cardia cancer than in the noncardia GC and should be further investigated.

In conclusion, our data suggest that identifying stem cell-like CTCs would provide more specific prognostic information regarding recurrence risk than merely detecting CTCs. Whether CTCs from GC patients embed stem cell-like characteristics still requires more extensive prospective studies. To our knowledge, this is the first report to identify the stem cell-like characteristics of CTCs and their prognostic significance in GC patients. Further molecular analysis of CTCs is needed to define possible targets for prevention and potentially treat micrometastasis to improve GC patient outcomes.

## Figures and Tables

**Table 1:** The relation between CTCs and clinicopathological data of GC patients.

Characteristic	Total	CTC		*χ* ^2^	*P*
		Positive	Negative		
Gender					
Male	27	19	8	3.025	0.122
Female	18	8	10		
Age					
≥60	29	20	9	2.732	0.122
<60	16	7	9		
Tumor location					
Proximal	32	21	11	1.46	0.317
Distant	13	6	7		
Histologic grade					
Poorly differentiated	23	13	10	0.237	0.763
Well differentiated	22	14	8		
Lymph node metastasis					
Positive	24	19	5	7.872	0.007
Negative	21	8	13		
Distant metastasis					
Positive	19	15	4	4.919	0.035
Negative	26	12	14		
TNM stage					
I-II	25	18	7	3.375	0.125
III-IV	20	9	11		
Recurrence					
Positive	19	15	4	4.919	0.035
Negative	26	12	14		

**Table 2:** The relation between CD44-positive CTCs and clinicopathological data of GC patients.

Characteristic	Total	CD44 + CTCs		*χ* ^2^	*P*
		Positive	Negative		
Gender					
Male	18	13	5	0.089	1
Female	9	6	3		
Age					
≥60	18	12	6	0.355	0.676
<60	9	7	2		
Tumor location					
Proximal	19	16	3	5.891	0.027
Distant	8	3	5		
Histologic grade					
Poorly differentiated	15	12	3	1.501	0.398
Well differentiated	12	7	5		
Lymph node metastasis					
Positive	16	14	2	5.527	0.033
Negative	11	5	6		
Distant metastasis					
Positive	13	12	1	5.787	0.033
Negative	14	7	7		
Recurrence					
Positive	14	13	1	7.052	0.011
Negative	13	6	7		

## Abbreviations

CTCs: Circulating tumor cells
GC: Gastric cancer
CSC: Cancer stem cell
CTSC: Circulating tumor stem cells
PBMCs: Peripheral blood mononuclear cells
PBS: Phosphate buffered saline
DAPI: 4,6-Diamino-2-phenylindole
LCA: Leukocyte Common Antigen.
